# What Patients Should Know

**Published:** 1899-12

**Authors:** A. T. Bigelow

**Affiliations:** St. Paul, Minn.


AMERICAN JOURNAL

—OF—

DENTAL SCIENCE.

VOL. XXXIII. BALTIMORE, DECEMBER, 1899. NO. 8.

### Selections.

ARTICLE I.

### WHAT PATIENTS SHOULD KNOW.

DR. A. T. BIGELOW, ST. PAUL, MINN.

The closing of the century is memorable for the attempts to right all wrongs, real or imaginary, of an individual, national or international character. There is a trend in the current of thought which marks an epoch that may be productive of benefit to toilers in many weary fields in many lands.

Consonant with human nature the general observation reverts to the special one's immediate surroundings and vocation.

The theme concerns all artists who are ambitious to carve their way to a niche in the temple of fame through sensitive dentine.

Generally, patients think they have the harder lot, possibly not considering that they sit only an hour or two, while the operator is on duty daily from five to seven hours; their trials are the most poignant, our duties on the drop by drop principle, the more wearing.

A glance backward discerns many a worthy brother early fallen by the wayside, and others within our personal knowledge whose fagged appearance shows too well the great nervous strain to which they are daily subjected.

Are not dentists as strong and vigorous at the outset as members of the other professions ?

However this may be, is it not a fact that their expectation of life as computed by the experienced actuary is the lowest of all.

It is not a difficult task to seek for the causes which are instrumental in undermining the constitution and abridging life's span.

The chair duties are specially wearisome in long-continued efforts, exposed to the 'unwholesome draught,' the back resembling a spiral, the lateral curvature greatly exaggerated, cramping the spinal cord and great sympathetic, thereby impeding the functions of all the vital organs.

When it is considered that the kindly handling of patients had taxed to the utmost the inventive genius, the strength, and it may be said the life of some of the brightest lights of the profession, is it not time that a treaty of more hearty and complete reciprocity be negotiated with the powers that be at the other end of the instrument ?

Could patients possess a better understanding of the proper condition in which to present themselves, and the desired etiquette of the dental chair, it would greatly facilitate the work, elevate the standard and prove a veritable boon to all concerned.

'Tis said, "the age is gone o'er when a man in all things be all." The times demand a separation or division of labor.

Our patrons cannot be expected to evolve from their inner consciousness a correct idea of the duties in the premises, but should look to us as specialists for all requisite information.

The instruction naturally begins with negatives—the things contra-indicated precedent to a sitting. No special characteristics will be noted in chair ordeals peculiar to men except the one that, as a rule, they endure pain less patiently and objurgate more forcibly than women.

In the main the suggestions offered apply equally well to both sexes unless otherwise designated.

Nervous ladies should not make an appointment to be met immediately after a reception, a fashionable "German," or any other trying social or domestic duty, but should so arrange that the date of sitting will find them at their physical best. The condition of mind also is of moment, for any violent emotion or mental perturbation speedily reac and leaves the body in a state of languor and lassitude.

A study to conserve nervous bone in some exceptional cases might be made with advantage.

The athlete, in order to triumph over an antagonist, subjects himself to a severe regimen, daily exercise, and a strict observance of hygienic laws. Caries may be justly considered one of the curses generated by high civilization, and a successful effort to abort the evil consequences a triumph which well repays exertion and self-denial.

The writer has had a good opportunity to note peculiarities of patients in service in several states and one territory, and it may not be amiss to portray a few characteristics which, to the expert, will reveal several species of the genus *un-ideal.*

They may be partially enumerated as follows, viz.:

Every motion made about the chair is regarded with a feline watchfulness. They hold one spell-bound, as it were, like the ancient mariner, "with their glittering eye."

These insist upon a digital as well as optical examination of everything within reach, not excepting the annealed gold. No instrument can be used without a close scrutiny, and anxious inquiries as to the supposed effect of their touch.

These are rich in stock expressions of disapprobation of all instruments and appliances, which they project into the ears of the long-suffering operator at short intervals.

These never remain posed even while an instrument is being changed, and start so suddenly and frequently that all work must be done on the "fly;" both flexors and extensors must be on duty at the same time to prevent accidents, necessitating double tension. This species, fortunately, are not plentiful, but when encountered, draw freely on the contents of the nerve cells of the luckless operator.

These affect a dismal groaning during the preparation of cavity. A strong individuality only saves the dentist from the sensation of gradual loss of identity in that of the accoucheur.

Usually young ladies, who amuse themselves and tantalize you by gazing full into your eyes to see the filling operation mirrored there.

These persist in the retention of a mirror in hand into which they look "many times and oft." Then there are those whom I will charitably not classify, who pretend to be hurt and make a great ado, under the mistaken idea that more care will be exercised, and who then go away and boast of their *finesse* to friends.

In some instances mothers essay to keep a child in lap, and young girls a pet dog in arms, but one must draw the line somewhere, and it may well be drawn at babies and pugs !

One sitting lingers in my remembrance even yet, although it occurred in the good old Bay State about twenty years ago. *She* was unmarried, of somewhat ripe experience, strong-minded, and, as I distinctly remember, *well armed.* The excavation was being proceeded with, a sensitive point she petulantly ejaculated "Oh!" and, by a quick lateral movement, jabbed me in the epigastric region with a sharp elbow. I involuntarily exclaimed "Ah!" The ohs and ahs during that mutually trying operation were as regular as the systole and diastole of our respective throbbing hearts. Therefore my dear old partner, Dr. I., attended to her dental needs, he being better protected than I by adipose tissue at the vulnerable point.

This will suffice for one phase of the subject, and I think all who have been in practice ten years or more will bear witness that the imagination has not been drawn upon for any of these types of the disingenuous, for was it not of this sort that Milton penned the distich for us ?

> "Of their doings great dislike declared,
> And testified against their ways."

Thus far the physical has mostly been considered, but there are other forces, psychic and mesmeric, which, though I do not pretend to explain, are nevertheless felt, and constitute a potent factor in the relations of agent and subject.

If there is solicitude or want of full confidence in the occupant of the chair, it is measurably reflected to the practitioner, who thus labors at a disadvantage, and for this reason first sittings are usually most trying, and should be made short and easy until the principle of cognition is established or temperamental differences adjusted.

The phrase "en rapport," borrowed from mesmerism, admirably expresses this idea. It might be paraphrased and applied to our art after this manner, as the condition or relation in which the patient expresses by word and action a willingness to be subjected to some discomfort, and pain if need be, to effect the desired object.

It is practically true there are some natures so antagonistic, one to another, that the slightest contact is repugnant. When this untuned condition exists the indication is strong to seek professional services in a more harmonious environment.

Too intense sympathy is also opposed to the exhibition of skill. An excellent dentist is known to me, who will not attempt to fill a tooth for his wife—said it was impossible for him to suitably do the work.

Another confessed inability to serve his near relatives, but had no scruples regarding his wife, dryly remarking "that he did not mind hurting her; she was no blood relation,"

A pen portrait of an ideal patron will now be presented. The lady comes promptly, binds her hair with a fillet of lace or veiling, and, if very abundant, lets it down altogether; is habited in a gown that is not injured by a drop of water; retains the pose given in the chair; closes, or partially closes her eyes, and is oblivious to our surroundings.

Given such an opportunity one is at his best, and can produce work, after its kind, as beautiful and enduring as the columns of Karnac or Grecian statuary.

Chair department is a severe touchstone of character and breeding. All the virtues possessed are brought out in relief—patience, resolution, fortitude, consideration for others, and withal the nameless grace with which the tact-person submits to the inevitable.

The reciprocal obligation between the parties in question might be compared to that of active and silent partners in business, equally interested in securing the success of the firm.

The adage, "Trifles make perfection, but perfection is no trifle," is markedly shown in our calling, as success hinges on a series of delicate manipulations, each of which must lead with careful precision to the end. A failure or lack of accuracy at any stage mars the work and vitiates our efforts.

The best dentistry no more than saves poor modern teeth, and any grade less than the best is bad.

It is of moment that our patrons should know and realize to the fullest extent that it matters little how skillfully cavities are filled, by what method or with what material; if the lesions are not on exposed surfaces the teeth must thereafter be kept *scrupulously clean* to secure a good degree of permanency.

Would that this fact could be engraved upon an ivory tablet and placed in every gentleman's pocket and on every lady's chatelaine.

Though the cavity may be hermetically sealed and the tooth theoretically restored to its pristine state, yet the conditions must be made more favorable by care and attention or the same destructive agents will again create havoc, especially in approximal and cervical regions.

Comparatively few of the non-professional have any adequate idea of the injurious consequences of calcic deposits upon the teeth. If it were feasible to show them, in mouths other than their own, the effects of this accumulation, demonstrating that all gum tissue in contact with it is more or less diseased, causing absorption of the alveoli, recession of the gums and consequent loosening of the whole denture, and let them catch the fetor or moldy odor, no second lesson should be needed to convince of the necessity for its frequent and thorough removal.

A glance into the eye-piece of a good microscope with a portion of "tartar" freshly taken from the mouth, on the slide, would afford conclusive evidence of its unwholesomeness.

It is not a pleasing thought to a refined person that a substance be allowed to remain in the oral cavity, which is a habitat for micro organisms.

Dentists still seem derelict in teaching the proper use of the tooth brush to their clintele, with the valuable adjuncts of waxed floss, a good dentifrice and some one of the many excellent antiseptic detergents now deemed indispensable.

It would be invidious to name any particular brush as superior to others, especially if patented with peculiarly shaped bristles, "a hole in the handle and in a yellow box."

If practitioners have not the time or inclination to impart oral instruction in the use of the necessary brush, then by all means give the patient one of the above which comes with the best printed directions extant for the acquirement of the polite art of scientific tooth brushing.

One practice has been made somewhat prominent of late, in a mercantile manner, calculated to deceive the unwary by false hopes of painless operations.

Hypodermic injections of anæsthetizing drugs may be necessitated in certain crisis and exigencies which arise in medical practice, but such procedure is not often advisable or sanctioned by good usage in our specialty.

Neither should powerful obtundents be often employed, for they lower the vitality of the tooth, and in some cases endanger the life of the pulp. I sometimes employ ethyl chloride as a *dernier resort* under sufficient restrictions, with satisfactory results. However, dryness, keen, high-tempered, well adapted instruments deftly handled contain more of promise to patrons, so far as safely minimizing pain is concerned, than all the medicaments of the pharmacopœia.

We are constantly called upon to treat those whose dental organs are in a chronic state of hyperesthesia; for such no rules can be formulated.

To successfully meet these cases one soon arrives at his wit's end, and much needs as a *sustainer* to draw on the peculiar characteristics of some worthies of the past and present, viz., the patience of Job, the skill of Tubal Cain, the finesse of Machiavelli and the all-around excellencies of champion Jeffries.

Young mothers are fortunate if in the care of a physician who, deeply versed in medical lore, has also some slight idea of dentistry. They will be informed that the period of gestation is specially destructive to the dental organs, not only from the liability to resorption of the lime salts, but also from harmful medicines, notably any preparation of iron, acting directly or secondarily upon tooth structure.

It is known that a diet rich in phosphates will, in a measure, prevent the resorptive process, but obstetricians assert that such food tends to solidify the bony framework of the fœtus, thereby increasing the difficulty and danger of parturition.

Here is presented an apparent dilemma in which eminent gynechologists assist us to determine what course to pursus by prescribing *plenty* of *fruit acids* thereby holding the lime salts of the food in solution and insuring a full and natural supply to the mother's circulation for the nourishment of the being *in utero.*

Certain credulities still linger, even among the most intelligent of the laity, notwithstanding the efforts to popularize dental knowledge, and among them none is more common than that once the "nerve is killed" no more pain can be caused by a necrosed tooth. When informed that many of the most aggravated and painful affections least amenable to treatment never occur until after the devitalization of the pulp, the fact is received with surprise, and in not a few instances with a positive admixture of doubt-

One experience of pulp treatment, alveolar abscess or pericementitis, however, usually serves as a spur to make the subject more watchful and heedful in future. The pain connected with the teeth which all, old and young, so much dread, is really a blessing in disguise, for there are scores of people who, but for it, would neglect remedial measures until too late for best results Nature has wisely placed within the dentine sensitive fibrils which, like a semaphore, signal to the sensorium of approaching danger.

Burke, in his essay on the Sublime and Beautiful, defines the difference between pleasure and delight, and avers that the former may arise spontaneously, while freedom from pain constitutes delight.

If our patrons could be fully persuaded to accept this scientific definition given by the eminent statesman and orator, it would greatly encourage them to endure some pain with fortitude, looking forward to an early, satisfactory and delightful termination.—*Items of Interest.*

